# Suspected levetiracetam drug-induced lupus in a child: A case report

**DOI:** 10.1177/2050313X261473997

**Published:** 2026-08-07

**Authors:** Fiona Landells, Jeanine McColl

**Affiliations:** 1 Division of Dermatology, University of Calgary, AB, Canada; 2 Division of Rheumatology, Department of Pediatrics, Alberta Children’s Hospital, Calgary, Canada

**Keywords:** anticonvulsants, drug-induced abnormalities, levetiracetam, pediatrics, systemic lupus erythematosus, case report

## Abstract

Drug-induced lupus erythematosus (DIL) is an uncommon condition that mimics systemic lupus erythematosus (SLE) and accounts for approximately 10%–12% of SLE cases. Numerous drugs have been implicated, most commonly hydralazine, procainamide, isoniazid, and certain antiepileptics. Levetiracetam has rarely been associated with SLE. We describe the first known possible pediatric case of suspected levetiracetam-induced lupus erythematosus. A previously healthy 6-year-old girl developed thrombocytopenia, neutropenia, anemia, generalized lymphadenopathy, and a petechial rash several months after initiating levetiracetam therapy for absence seizures. Extensive infectious and oncologic investigations, including a bone marrow biopsy and an excisional lymph node biopsy were unremarkable. Given the temporal association with levetiracetam exposure, the presence of clinical features consistent with DIL, and a serological pattern suggestive of DIL, the drug was discontinued, and the patient was treated with corticosteroids, intravenous immunoglobulin, and hydroxychloroquine. She demonstrated rapid clinical and biochemical improvement. Although distinguishing DIL from early SLE remains challenging and no definitive diagnostic criteria exist, this case met several proposed features of DIL and scored as a possible adverse drug reaction on the Naranjo scale. This report expands the spectrum of levetiracetam-associated adverse effects and highlights the need for clinicians to consider DIL in pediatric patients even when receiving drugs with generally favorable safety profiles.

## Introduction

Drug-induced lupus erythematosus (DIL) is a rare condition in which exposure to a drug results in systemic symptoms resembling those of systemic lupus erythematosus (SLE). DIL is believed to represent approximately 10%–12% of cases of SLE.^1^ Isoniazid, hydralazine, procainamide, antiepileptics including phenytoin and carbamazepine, tumor necrosis factor inhibitors, and herbal supplements have been implicated in DIL.^1,2^

Although clinically similar to SLE, the symptoms associated with DIL are typically milder than those in SLE and rarely affect the central nervous system or kidneys.^2^ The symptoms most frequently observed in DIL in children are fever, arthralgia, rash, and arthritis. Cutaneous features of SLE such as alopecia, oral ulcers, malar rash, and photosensitivity are less common.^2^ Although SLE predominantly affects females, DIL does not demonstrate a predilection toward either sex.^2^ The autoantibody profile in DIL differs from that seen in SLE, with antihistone antibodies being reported in up to 95% of those with DIL while antidouble stranded DNA (anti dsDNA) antibodies and antiextractable nuclear antigen antibodies are less frequently observed.^2^ There are no accepted diagnostic criteria for DIL, making diagnosis challenging. The temporal relationship between initiation of a drug and the presentation of symptoms and the cessation of these symptoms with termination of the drug is the strongest indicator of DIL.^2^

A search of the medical literature was performed using the MEDLINE and Embase databases in June 2026 using the search terms “Drug-induced lupus,” “DIL,” “Leveracitam induced lupus erythematosus,” and “antiepileptic induced lupus.” We examined the literature from 1900 until present date. Upon review of the literature there have not been any conclusive reports of levetiracetam-induced lupus; however, there is one potential case reported in an adult patient^1^ which was not conclusive. This is the first published report of a case of possible levetiracetam-induced lupus in a child. Informed written consent was obtained for this case report.

## Case

A previously healthy 6-year-old female with no significant neonatal or family history was diagnosed with absence seizures, at which point ethosuximide was initiated. Two weeks after starting this drug, she developed an erythematous urticarial pruritic maculopapular rash to the cheeks, arms, and abdomen in keeping with a severe cutaneous adverse reaction (SCAR) versus reactive infectious mucocutaneous eruption (RIME). Two months later, she was switched to Teva-levetiracetam immediate release tablets at 250 mg twice daily (25 mg/kg/day). There is no family history of SCAR. Six months after the seizure diagnosis and 4 months after starting levetiracetam, she presented to hospital with cervical, axillary, and inguinal lymphadenopathy, mucosal bleeding, and a petechial rash. She was found to have thrombocytopenia with platelets of 1 × 10^9^/L. Of note, her platelets were normal (298 × 10^9^/L) when she was diagnosed with absence seizures with a slight decrease (136 × 10^9^/L) after the SCAR, they had been within normal range (208 × 10^9^/L) the month prior to this presentation to hospital. On exam, she appeared well but did have hyper-pigmented patches scattered on her arms, abdomen, and legs secondary to her previous SCAR as well as a generalized petechial rash. There was palpable cervical, axillary, and inguinal lymphadenopathy, which was soft and mobile. She did not have arthritis.

Three days after admission, she developed neutropenia (neutrophils of 0.5 × 10^9^/L) and anemia (hemoglobin of 99 g/L). Her baseline hemoglobin had previously been stable at 130 g/L. An extensive workup for hematologic malignancy including a bone marrow biopsy and excisional lymph node biopsy was noncontributory. An infectious workup was unremarkable. She was found to have positive antinuclear antibodies (ANA) with a titer of >1:640, positive ribonucleoprotein antibodies (anti-RNP) of 5.6 kIU/L, positive anti-dsDNA antibodies were low positive at 13 kIU/L, and positive antihistone antibodies at 7.1 kIU/L. Her anti-Smith, SS-A52, SS-A60, and SS-B antibodies, and antiphospholipid antibodies were all negative. Complements, C3 and C4 were 1.32 and 0.10 g/L, respectively. The erythrocyte sedimentation rate (ESR) was 110 mm/h, and she had proteinuria with a urine protein/creatinine ratio of 280.33 mg/mmol. Additionally, her serum creatinine was 31 µmol/L, her serum albumin was 36 g/L, and her urinalysis showed protein greater than 3.0 g/L with hyaline casts of 3–5 per low power field.

She was initially treated with intravenous immunoglobulin at 2 grams per kilogram (g/kg) IV for 2 days, 2 days apart, and methylprednisolone at 2 mg/kg IV daily for 2 days. Given the medication history and presentation, a potential diagnosis of SLE versus levetiracetam-induced lupus was made. She had not been on any other medications, other than ethosuximide, prior to this current presentation to hospital. She was started on a 4-month oral prednisone taper starting at 2 mg/kg daily, and oral hydroxychloroquine at 100 mg (5.5 mg/kg) daily. The levetiracetam was discontinued 16 days after admission. She quickly improved with this treatment regimen with normalization of the neutrophils to 0.9 × 10^9^/L within 6 days of stopping levetiracetam. The hemoglobin normalized to 116 g/L within 3 weeks. The anti-dsDNA, which had increased up to 80 during the first 2 week in hospital, quickly dropped down to 8 kIU/L within a month of stopping the drug. Additionally, her elevated protein/creatinine ratio decreased to 28.57 mg/mmol. Due to the rapid improvement in her renal involvement and her normal serum creatinine, nephrology did not believe there to be evidence of lupus nephritis, and a renal biopsy was contraindicated given her thrombocytopenia. Upon follow-up, she has now discontinued hydroxychloroquine and prednisone, her ESR, urinalysis, urine protein/creatinine ratio, anti-dsDNA, and antihistone antibodies remain normal. There has been no recurrence of her symptoms.

## Discussion

Many drugs have been implicated in DIL with clinical symptoms that resemble those of SLE. Symptoms of DIL most often occur within months to years of exposure to the offending drug; however, there have been reports of the development of DIL within days or weeks of exposure.^3^

Levetiracetam is a broad-spectrum antiepileptic which is generally well tolerated and effective. Although not approved by Health Canada for use in the pediatric population, as per the Food and Drug Administration, standard dosing in pediatric patients between 4 and 16 years of age, starts at 20 mg/kg/day divided in two equal doses per day. This can then be up titrated by 20 mg/kg/day every 2 weeks until a maximum dose of 60 mg/kg/day is reached.^4^ Although its mechanism of action is not completely understood, its antiepileptic properties are thought to be related to its ability to bind to synaptic vesicle protein 2A (SV2A), a protein found on the membrane of secretory vesicles involved in calcium-dependent neurotransmitter release.^5^ The improved safety profile of levetiracetam when compared with other antiepileptics is felt to be related to this unique mechanism of action.^6^ Common adverse effects associated with this drug include headaches, vomiting, drowsiness, and worrisome psychological symptoms such as suicidal ideation and depression. Severe adverse events reported include cytopenia, SCARs—Stevens–Johnson syndrome, toxic epidermal necrolysis, and drug reaction with eosinophilia and systemic symptoms—as well as anaphylaxis and acute kidney injury.^7–9^ There has only been one case reported of levetiracetam-induced lupus in which the patient experienced cutaneous symptoms. Given the difficulty in differentiating DIL from SLE, it is hypothesized that levetiracetam-induced lupus is more common in practice.^1^

Lupus with neuropsychiatric involvement is more common in childhood SLE than adult onset SLE with a reported incidence ranging from 14% to 95%. Seizures are present in 9.5%–84.4% of children with neuropsychiatric SLE and are often associated with antiphospholipid antibodies.^10–12^ This patient’s antiphospholipid antibodies were negative. The previous diagnosis of absence seizures in this patient is thought to be unrelated to her disease presentation as she did not have other clinical SLE features at seizure onset and SLE features resolved with treatment and discontinuation of the drug.

The mechanism of DIL is not well understood. It is thought to be secondary to a complex interplay between immune response, drug metabolism, and genetics. One theory is many drugs that cause DIL are converted to reactive metabolites via oxidative processes from leucocytes. This results in neoantigen formation that triggers an immune response.^13,14^ Interestingly, levetiracetam has been found to act as an antioxidant suggesting a neuroprotective role in epilepsy models.^15,16^ It has minimal oxidative metabolism, with oxidation accounting for less than 2.5% of the dose in humans as it is not primarily metabolized via the cytochrome P450 pathway in the liver.^17^ This may explain why DIL has been so rarely reported with levetiracetam.

DIL is rare in pediatric populations. The majority of data on this condition is from the literature on adult patients. A review by Kaya Akca et al.^2^ in 2024 was performed on DIL specifically in children. These authors reported that fever, arthralgia, rash, and arthritis were the most commonly reported symptoms in children with DIL. Thrombocytopenia and lymphadenopathy were less commonly reported, these affected 4.6% and 16.9% of patients, respectively. In terms of the serological profile of these patients, ANA positivity was reported in 93.5% of cases, anti-dsDNA antibodies were reported in 57.4% of cases, and antihistone antibodies were reported in 72.2% of cases.^2^ This is similar to the adult literature which reports that anti-dsDNA antibodies in DIL are rare while antihistone antibodies are present in greater than 90% of DIL cases (conversely these occur in only 50% of SLE cases).^3^ The presence of low-titer positive anti-dsDNA antibodies with antihistone antibodies is thought to be highly suggestive of DIL versus SLE.^3^ In our case, the patient had low titers of anti-dsDNA antibodies, with associated positive antihistone antibodies which again, is consistent with DIL.

Our patient did have some features which were atypical for DIL including cytopenias, anti-dsDNA positivity, anti-RNP positivity, generalized lymphadenopathy, and an elevated urine protein/creatinine ratio. Although she did have clinical evidence of renal involvement initially upon presentation, this resolved less than 1 month after cessation of levetiracetam and her serum creatinine and serum albumin were normal throughout her admission to hospital. Additionally, the presence of antibodies, lymphadenopathy, and cytopenias all improved rapidly within 1 month of cessation of levetiracetam. These abnormalities have continued to remain normal since she stopped treatment with hydroxychloroquine and prednisone, which further support that this renal involvement was secondary to DIL and not SLE.

Although DIL is a well-established condition, there are currently no accepted diagnostic criteria to help differentiate this condition from SLE. One set of criteria was proposed by Borchers et al. in 2007 which requires the presence of 4 points, one for each of the following:

(a) exposure to a specific drug for a sufficient amount of time,
(b) the presence of at least one symptom consistent with SLE,
(c) no previous history of symptoms consistent or suggestive of SLE prior to initiation with therapy, and
(d) the resolution of the patient’s symptoms once the drug has been discontinued.^3^

In our case, the patient does fulfill these criteria for DIL. She developed several symptoms consistent with SLE—thrombocytopenia, neutropenia, proteinuria, and the presence of multiple autoantibodies. Infectious and oncological causes of these symptoms were ruled out. After several months of exposure to levetiracetam, her symptoms did resolve once levetiracetam was discontinued and once she was treated with systemic corticosteroids and hydroxychloroquine. Although this patient had experienced a prior possible SCAR while being treated with ethosuximide, this medication was not suspected as the causative agent in her presentation of DIL. Ethosuximide has been discontinued nearly 6 months prior to this patient’s onset of DIL symptoms, whereas she was actively taking levetiracetam at the time she developed symptoms. It was postulated that as she did have a prior history of drug-induced SCAR, she may be more at risk for developing further drug-induced hypersensitivity reactions or even autoimmune disease. In fact, a study by Lin et al.^18^ reported an incidence of autoimmune disease as high as 2.2% in patients with previous drug-induced SCAR versus 0.1% in patients without prior history of SCAR. Additionally, the exact etiology of her severe cutaneous reaction was not completely clear as this occurred outside of our medical authority, and the medical records were not available to us. From history, our understanding was that RIME was also a possible etiology for her rash which is a reaction secondary to infection and not a drug.

A Naranjo adverse drug probability scale was completed for this case which yielded a score of 3.^19^ Two points were obtained for the chronological relationship between the patient’s symptoms and the introduction of levetiracetam, 1 point was obtained for the patient’s symptoms resolving after the discontinuation of levetiracetam, 1 point was lost as SLE was a potential alternative diagnosis. Finally, 1 point was obtained as she did have objective findings which confirmed an adverse event—the presence of ANA, and antihistone antibodies. This score of 3 determined there was a possible adverse drug reaction.^19^ Of note, this patient had no history consistent with prior symptoms of SLE, with the caveat that she did develop childhood absence seizures several months prior to her hospitalization. This raises the question as to whether or not these seizures were a prior symptom of SLE, which would be quite unusual as an initial presentation with no other clinical or biochemical features of SLE. Re-exposure to levetiracetam would have confirmed this case as DIL or SLE, this was thought to be unethical and was not pursued. Re-introduction to an offending agent in DIL often results in more severe reactions.^3^

Common first-line agents in the treatment of absence seizures in children, include ethosuximide, valproic acid, and lamotrigine. Ethosuximide and valproic acid have been found to be more effective than lamotrigine, although valproic acid often has more side effects (weight gain, hair loss, and attention challenges). Ethosuximide is favored as it has less adverse effects on attention.^20^ All three agents have been implicated in DIL.^21–23^ The literature is mixed regarding the efficacy of levetiracetam.^23^

**Figure 1.**
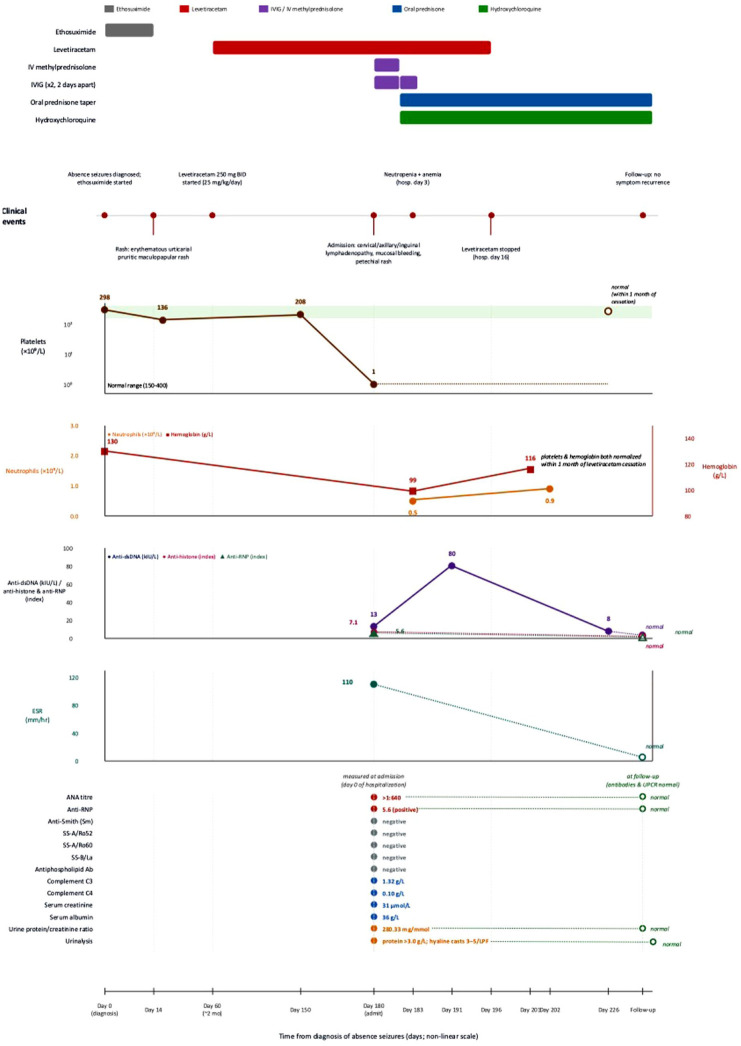
Timeline of possible levetiracetam-induced lupus erythematosus.

We share this case as the first reported instance of levetiracetam-induced lupus erythematosus in a pediatric patient to emphasize the importance of assessing the safety of all antiepileptics as well as to remind general practitioners, pediatricians, dermatologists, and rheumatologists to keep DIL on their differential diagnosis (Figure 1).
